# Retinal and Choroidal Vascular Perfusion and Thickness Measurement in Diabetic Retinopathy Patients by the Swept-Source Optical Coherence Tomography Angiography

**DOI:** 10.3389/fmed.2022.786708

**Published:** 2022-03-18

**Authors:** Tingting Liu, Wei Lin, Genggeng Shi, Wenqi Wang, Meng Feng, Xiao Xie, Tong Liu, Qingjun Zhou

**Affiliations:** ^1^Eye Hospital of Shandong First Medical University (Shandong Eye Hospital), Jinan, China; ^2^State Key Laboratory Cultivation Base, Shandong Provincial Key Laboratory of Ophthalmology, Shandong Eye Institute, Qingdao, China; ^3^Shandong First Medical University and Shandong Academy of Medical Sciences, Qingdao, China; ^4^School of Ophthalmology, Shandong First Medical University, Jinan, China; ^5^School of Basic Medicine, Shandong First Medical University and Shandong Academy of Medical Science, Jinan, China; ^6^The First Clinical Medical College of Shandong University of Traditional Chinese Medicine, Jinan, China; ^7^Department of Medicine, Xizang Minzu University, Xianyang, China; ^8^Qingdao Eye Hospital of Shandong First Medical University, Qingdao, China

**Keywords:** diabetic retinopathy, swept-source optical coherence tomography angiography, deep capillaries, choroidal vascular perfusion, retinal vascular perfusion

## Abstract

**Purpose:**

To observe the changes in retinal and choroidal microstructures in patients with different stages of diabetic retinopathy (DR) and to evaluate the vascular perfusion of retina and choroid retinal thickness, retinal and choroidal vessel density by the swept-source optical coherence tomography angiography (SS-OCTA).

**Methods:**

Subjects were divided into three groups: healthy control group (30 cases, 51 eyes), non-proliferative diabetic retinopathy (NPDR, 42 cases, 71 eyes) and proliferative diabetic retinopathy (PDR, 31 cases, 53 eyes). The area of the foveal avascular zone (FAZ), retinal and choroidal vascular perfusion, and the deep vascular complexes, including the intermediate capillary plexus (ICP) and deep capillary plexus (DCP) within the radius of 3, 6, 9, and 12 mm around the fovea were measured by SS-OCTA.

**Results:**

Compared with the healthy control group, DR patients presented significantly increased fovea avascular area, while vessel density (VD) in the ICP and DCP, vascular perfusion rate, and the retinal thickness were considerably decreased. There were significant differences in the retinal thickness, ICP, and DCP vessel densities between the control and NPDR groups, or control and PDR groups, or PDR and NPDR groups. The deep vascular perfusion rate also significantly differed between the control and PDR groups, but there was no significant difference between the PDR and NPDR groups. The choroidal perfusion exhibited significant differences across different areas and amongst the three groups. Furthermore, the choroidal thickness showed a significant difference between the PDR and NPDR groups.

**Conclusion:**

Our results showed significant differences in the area of the avascular fovea and the VD of deep vascular complexes between DR patients and healthy control subjects. Moreover, there were significant differences in retinal VD, especially in the deep-retinalregions, choroidal perfusion, and the volume of large vessel choroid in DR patients with different degrees of disease severity. Notably, SS-OCTA provided in-depth information for detecting the potential VD damage in DR patients caused by a multitudeof factors.

## Introduction

Diabetic retinopathy (DR) is one of the most detrimentallong-term diabetes complications that damage fundus retinal microvasculature complications in DM patients (1). Although mild DR complications can be cured by proper management of the diabetic condition, DR induced gradual deterioration of vision is a common risk factor for permanent blindness in DM patients (2–5). Non-proliferative DR (NPDR) is characterized by asymptomatic microvascular changes or minute leaks in the retina, which ultimately develops into proliferative DR (PDR) or diabetic macular edema (DME) with the gradual progression of DM (6, 7). Therefore, early diagnosis of DR and precise estimation of the retinal damage already caused by DM are critical for the prevention of aging-independent retinal damage and complete blindness (5, 8).

The ultra-wide-angle imaging of the swept-source optical coherence tomography angiography (SS-OCTA) has multiple advantages like faster scanning speed, higher signal intensity, and deeper penetration depth. This technique is also used to detect early phases of retinal microvascular damages in DM patients, It did not lead to obvious visual impairment (9–11). Since SS-OCTA operates at 1,050 nm wavelength light sources, so it is less susceptible to energy attenuation in the fundus and offers better penetrance capacity. And they are very important factors for accurate diagnosis and clinical researches of DR. Studies have shown that SS-OCTA provides consistent results with OCTA in the diagnosis of DR (12). Moreover, compared with the spectral-domain optical coherence tomography angiography (SD-OCTA), SS-OCTA has a larger inspection scope, higher frequency, and faster scanning speed. Thus, SS-OCTA can more accurate and real-time monitor the peripheral retinal VD lesions of DR patients (13–15).

SS-OCTA can also present clearer choroidal images, automatically detect and analyze choroidal thickness, reduce manual measurement errors, and improve the efficiency of choroidal observation and diagnosis. With increasing scan depth, the possibility of signal attenuation will be further reduced, which is suitable for imaging larger fields and distinct observation of the vitreous body, retina, choroid, and sclera for detailed study of the fundus in DM patients (16). Such unique advantages of SS-OCTA method have immense clinical significance for early diagnostic confirmation of lesion extent in DME.

In this study, the SS-OCTA technique was applied to observe and quantitatively analyze the retinal and choroidal microvascular parameters like VD, capillary network, and morphological alterations and compare the foveal avascular zone (FAZ) in different macular regions between healthy subjects and patients with NPDR or PDR. Therefore, this study would provide critical diagnostic guidelines for DM patients with different degrees of retinal damage.

## Methods

### General Information

This study recruited 73 patients who were Type 2 diabetes mellitus (T2DM, lesions identified in 124 eyes). Among them 31 PDR patients including 24 males (40 eyes) and 7 females (13 eyes), 42 NPDR patients including 29 males (51 eyes) and 13 females (20 eyes). And 30 healthy control subjects without a history of diabetes, including 12 males (17 eyes) and 18 females (34 eyes). All subjects involved in our study have been recruited from the Department of Fundus Medicine of Shandong Eye Hospital in 2020–2021.

### Inclusion and Exclusion Criteria for Participants

The inclusion criteria were as follows: (1) the symptoms of the patients met the diagnostic criteria of DR with T2DM (17); (2) except for DM, presence of no other systemic diseases; (3) except DR, the occurrence of no other eye diseases. The exclusion criteria included: (1) unable to cooperate with the examination due to nystagmus or other reasons; (2) refractive interstitial opacity, poor SS-OCTA imaging quality. All patients were examined by the best corrected visual acuity (BCVA), using the international standard chart of vision, and the logarithm of the minimum angle of resolution (LogMAR).

### SS-OCTA Examination

All the subjects were examined by SS-OCTA (SS-OCTA, VG200D, SVision Imaging, Ltd., China) (18), with the scanning mode 3 × 3 mm for FAZ measurement and to obtain the fundus images with the central area of 3 × 3 mm around the fovea of the macula. The data of 12 × 12 mm area centered on the fovea were obtained with 1,024 × 1,024 B-scans, 12 × 12 mm SS-OCTA images were captured with the commercial VG200 SS-OCTA device (19).

Furthermore, SS-OCTA also may not show leakage. However, these artifacts do not affect the analysis of non-perfused regions. At the same time, these artifacts are also subtle. We try to avoid these problems in methods and eliminate artifacts as much as possible. We manual segmentation when machine had segmentation errors.

### Statistical Analysis

The data obtained were statistically analyzed by SPSS 22.0 software. Our data included the basic demographic information of the subjects, the lesion area, perimeter, acircularity index (AI) and VD of FAZ. We also evaluated the changes of retinal and choroidal VD, vascular perfusion, and retinal thickness of the deep vascular complex in the circular radii of 3, 6, 9, and 12 mm around the fovea. We used an independent sample *t*-test to analyze the data. The detection level threshold was set at α = 0.05 and calculated as mean ± Standard Deviation (mean ± SD). Results with *p* < 0.05 were considered a statistically significant difference.

## Results

### Characteristics of the Patients With DR

In this study, we investigated 30 cases (51 eyes) healthy control subjects and 73 cases DR patients (124 eyes), which included two groups, namely NPDR (71 eyes) and PDR group (53 eyes). And 73 DR patients also suffered from T2DM. There were no significant differences in gender between patients with NPDR and PDR (*x*^2^ = 0.628, *p* = 0.596). Concurrently, for age, there were no significant differences between the healthy control group (age range: 23–72 year; mean ± SD: 53.97 ± 11.43) and PDR (age range: 32–72 year; mean ± SD: 54.71 ± 10.17, *p* = 0.789) or NPDR (age range: 34–79 year; mean ± SD: 58.26 ± 9.93, *p* = 0.094) group, and there was also no significant difference between PDR and NPDR (*p* = 0.139) group. The BCVA of healthy controls was 0.01 ± 0.03 LogMAR. The BCVA of NPDR patients was 0.3 ± 0.24 LogMAR, and PDR patients was 0.6 ± 0.50 LogMAR, our results suggested that the vision of PDR was worse than that of NPDR group (*p* < 0.05). The average intraocular pressure (IOP) of patients was also decreased to 16 ± 1.3 mmHg (the normal range was 12–21 mmHg), there was no significant difference in IOP between NPDR and PDR groups.

### FAZ Measurement

We compared the area, perimeter, AI, and VD of the fovea FAZ between DR patients and healthy controls, as shown in Table 1 and Supplementary Figure S1. The results showed that the area, perimeter and VD of FAZ in PDR patients were significantly increased, compared to the healthy control group (FAZ area: 0.38 ± 0.08 vs. 0.49 ± 0.23, *p* = 0.030 < 0.05; perimeter: 2.58 ± 0.32 vs. 3.08 ± 0.90, *p* = 0.016 < 0.05; VD: 42.70 ± 5.37 vs. 34.80 ± 5.53, *p* = 0.000 < 0.001). The perimeter, AI, and VD of NPDR subjects were also increased significantly, compared to that of the healthy control group (FAZ perimeter: 2.58 ± 0.32 vs. 2.93 ± 0.58, *p* = 0.029 < 0.05; AI: 0.71 ± 0.09 vs. 0.64 ± 0.11, *p* = 0.010 < 0.05; VD: 42.70 ± 5.37 vs. 37.36 ± 5.96, *p* = 0.001). However, these indices were not significantly different between the NPDR and PDR groups. Hence, these results suggest that the rate of changing of FAZ may be related to the advancement of DR pathology, as observed as differential FAZ pathobiology between NPDR and PDR patients, compared to the healthy control subjects.

**Table 1:** SS-OCTA evaluated the FAZ with centered on the macular fovea in control eyes, PDR eyes, NPDR eyes (mean ± SD, *p*-value).

**Zone**	**Control** **(*n* = 43)**	**PDR** **(*n* = 23)**	**NPDR** **(*n* = 17)**	**Control vs. PDR** **(*p/t* value)**	**Control vs. NPDR** **(*p/t* value)**	**PDR vs. NPDR** **(*p/t* value)**
Area (mm^2^)	0.38 ± 0.08	0.49 ± 0.23	0.44 ± 0.16	0.030^*^/−2.304	0.131/−1.578	0.429/0.800
Perimeter (mm)	2.58 ± 0.32	3.08 ± 0.90	2.93 ± 0.58	0.016^*^/−2.577	0.029^*^/−2.353	0.533/0.629
AI	1.43 ± 0.19	1.62 ± 0.43	1.61 ± 0.30	0.050^*^/−2.521	0.029^*^/−2.343	0.921/0.100
VD (mm^−1^)	42.70 ± 5.37	34.80 ± 5.53	37.36 ± 5.96	0.000^***^/5.619	0.001^***^/3.358	0.170/−1.399

* p ≤ 0.05, there is a statistical difference; ^**^p ≤ 0.01,

*** *p ≤ 0.001, thereis a significant difference*.

### Retinal Vascular Complexes

**Figure 1:**
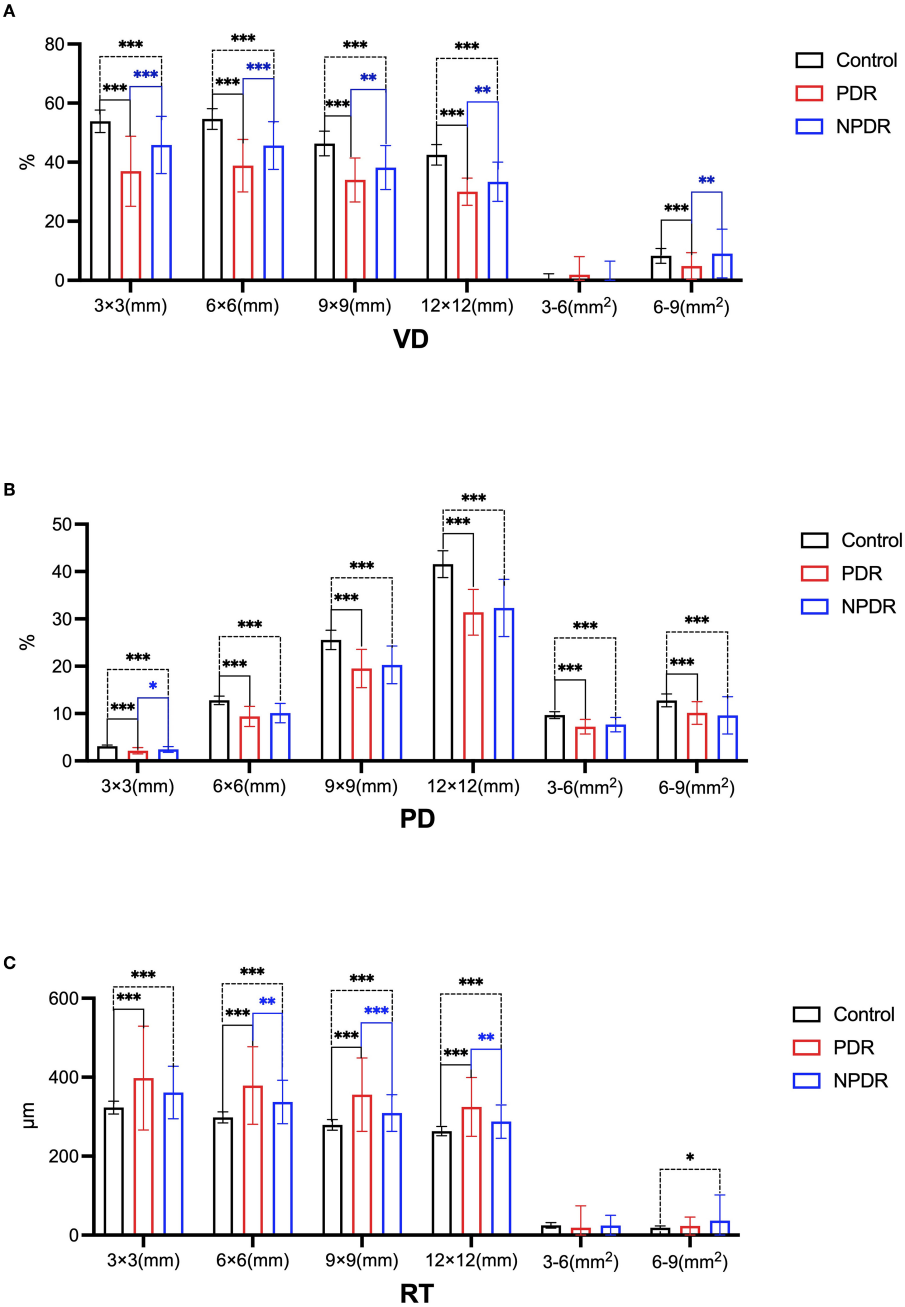
The changes of the deep vascularcomplex in healthy control group, PDR group and NPDR group by SS-OCTA (*p ≤ 0.05,there is a statistical difference.**p ≤ 0.01, ***p ≤ 0.001, there is a significant difference). **(A)** Comparison of VD in 3 × 3 (mm), 6 × 6 (mm), 9 × 9 (mm), 12 × 12 (mm), 3–6 (mm^2^), and 6–9 mm (mm^2^) in the healthy controls, PDR and NPDR groups. **(B)** Comparison of PD in 3 × 3 (mm), 6 × 6 (mm), 9 × 9 (mm), 12 × 12 (mm), 3–6 (mm^2^), and 6–9 mm (mm^2^) in the healthy controls, PDR and NPDR groups. **(C)** Comparison of RT in 3 × 3 (mm), 6 × 6 (mm), 9 × 9 (mm), 12 × 12 (mm), 3–6 (mm^2^), and 6–9 mm (mm^2^) in the healthy controls, PDRand NPDR groups.

Next, we compared the VD, perfusion density (PD), and retinal thickness (RT) of deep vascular complexes in the circular radius at the center of the fovea of DR patients and healthy controls, as shown in Table 2 and Figure 1.

**Table 2:** SS-OCTA evaluated the deep vascular complex with different radii centered on the macular fovea in control eyes, PDR eyes, NPDR eyes (mean ± SD, *p*-value).

**Variable**	**Radii**	**Control**	**PDR**	**NPDR**	**Control vs. PDR**	**Control vs. NPDR**	**PDR vs. NPDR**
VD		***n*** **=** **51**	***n*** **=** **52**	***n*** **=** **46**	**(*****p/t*** **value)**	**(*****p/t*** **value)**	**(*****p/t*** **value)**
	3 ×3 mm	53.85 ± 3.81	36.94 ± 11.86	45.84 ± 9.70	0.000^***^/9.781	0.000^***^/5.250	0.000^***^/−4.083
	6 ×6 mm	54.63 ± 3.53	38.85 ± 8.88	45.61 ± 8.08	0.000^***^/11.892	0.000^***^/6.993	0.000^***^/−3.941
	9 ×9 mm	46.34 ± 4.16	33.99 ± 7.47	38.19 ± 7.47	0.000^***^/10.605	0.000^***^/6.425	0.006^**^/−2.784
	12 ×12 mm	42.49 ± 3.47	30.01 ± 4.59	33.36 ± 6.64	0.000^***^/14.664	0.000^***^/7.859	0.009^**^/−2.671
	6–3 mm^2^	−0.78 ± 3.01	1.91 ± 6.13	0.23 ± 6.30	0.235/−1.191	0.596/0.55	0.185/1.336
	9–6 mm^2^	8.29 ± 2.52	4.87 ± 4.52	9.08 ± 8.22	0.000^***^/4.765	0.536/−0.651	0.002^**^/−3.089
PD		***n*** **=** **51**	***n*** **=** **53**	***n*** **=** **70**	**(*****p/t*** **value)**	**(*****p/t*** **value)**	**(*****p/t*** **value)**
	3 × 3 mm	3.12 ± 0.24	2.14 ± 0.66	2.42 ± 0.61	0.000^***^/10.071	0.000^***^/8.805	0.019^*^/−2.356
	6 ×6 mm	12.80 ± 0.89	9.39 ± 2.14	10.09 ± 2.04	0.000^***^/10.679	0.000^***^/9.902	0.068/−1.845
	9 ×9 mm	25.58 ± 2.04	19.51 ± 4.05	20.29 ± 3.96	0.000^***^/9.496	0.000^***^/9.487	0.299/−1.044
	12 ×12 mm	41.56 ± 2.82	31.39 ± 4.84	32.31 ± 6.06	0.000^***^/12.292	0.000^***^/10.778	0.405/−0.837
	6–3 mm^2^	9.68 ± 0.73	7.24 ± 1.55	7.67 ± 1.53	0.000^***^/10.298	0.000^***^/9.592	0.131/−1.521
	9–6 mm^2^	12.79 ± 1.36	10.13 ± 2.39	9.62 ± 3.93	0.000^***^/7.014	0.000^***^/6.237	0.410/0.828
RT		***n*** **=** **51**	***n*** **=** **53**	***n*** **=** **70**	**(*****p/t*** **value)**	**(*****p/t*** **value)**	**(*****p/t*** **value)**
	3 ×3 mm	323.01 ± 16.03	398.71 ± 132.65	361.49 ± 66.14	0.000^***^/−4.123	0.000^***^/−4.683	0.065/2.038
	6 ×6 mm	298.10 ± 14.05	380.01 ± 98.86	337.68 ± 55.08	0.000^***^/−5.969	0.000^***^/−5.759	0.006^**^/2.805
	9 ×9 mm	279.22 ± 13.32	357.07 ± 93.38	309.68 ± 46.48	0.000^***^/−6.006	0.000^***^/−5.13	0.001^***^/3.383
	12 ×12 mm	263.31 ± 11.77	326.14 ± 74.85	287.96 ± 42.34	0.000^***^/−5.568	0.000^***^/−4.437	0.003^**^/3.092
	6–3 mm^2^	24.90 ± 7.03	18.7 ± 55.93	23.82 ± 24.99	0.427/0.786	0.731/0.301	0.537/−0.682
	9–6 mm^2^	18.88 ± 4.32	22.94 ± 22.8	36.85 ± 65.61	0.209/−1.248	0.025^*^/−2.284	0.143/−1.476

* *p ≤ 0.05, there is a statistical difference*;

** *p ≤ 0.01*,

*** *p ≤ 0.001, there is a significant difference*.

The mean VD in both the NPDR and PDR groups were lower than that of the healthy control group, with significant differences in 3 mm (3 × 3 mm), 6 mm (6 × 6 mm), 9 mm (9 × 9 mm), and 12 mm (12 × 12 mm) captures between the healthy control and NPDR group or PDR group (Table 2; Figure 1). And the mean VD in 3–6 mm^2^ and 9–6 mm^2^ captures of the PDR were l± ± over than that of control subjects. Moreover, the mean values of VD in 3 mm (3 × 3 mm), 6 mm (6 × 6 mm), 9 mm (9 × 9 mm), 12 mm (12 × 12 mm), and 9–6 mm^2^ captures of the PDR lesions were also lower than that of the NPDR group. There were significant differences in mean values of PD between NPDR or PDR and control subjects in all captures, with the lower mean PD in DR patients compared to control subjects (Table 2; Figure 1). But the difference between mean values of PD in the NPDR and PDR groups was not significant. On the other hand, the mean value of RT in DR patients (both NPDR and PDR) was significantly higher than that of control subjects, in 3 mm (3 × 3 mm), 6 mm (6 × 6 mm), 9 mm (9 × 9 mm), and 12 mm (12 × 12 mm) captures. And the mean values of RT in 3 mm (3 × 3 mm), 6 mm (6 × 6 mm), 9 mm (9 × 9 mm), 12 mm (12 × 12 mm) captures of the PDR group were significantly higher than that of NPDR group.

These data indicated that VD, PD, and RT in NPDR and PDR patients have different degrees of damage, especially in VD and RT. Notably, RT and VD had reciprocal correlations between the PDR and NPDR groups, suggesting that PDR might be characterized by hyperplasia. Moreover, the mean retinal PD was significantly lower in DR patients (in both NPDR and PDR patients) compared to control subjects.

### Choroidal Vascular Complexes

Table 3 exhibits the values of choroidal perfusion (CP), choroidal vascularity index **(**CVI), choroidal vascularity volume (CVV), and choroidal thickness (CT) in the circular radius at the center of the fovea of DR patients and healthy controls. The results showed that the CT was significantly increased in NPDR and PDR patients than in control subjects (Table 3; Supplementary Figure S2). There was significant differences in 3–6 mm (3–6 mm^2^) captures of CT between the PDR and NPDR groups.

**Table 3:** SS-OCTA evaluated the choroidal indices with different radii centered on the macular fovea in control eyes, PDR eyes, NPDR eyes (mean ± SD, *p*-value).

**Variable**	**Radii**	**Control**	**PDR**	**NPDR**	**Control vs. PDR**	**Control vs. NPDR**	**PDR vs. NPDR**
**CP**		***n*** **=** **51**	***n*** **=** **53**	***n*** **=** **69**	**(*****p/t*** **value)**	**(*****p/t*** **value)**	**(*****p/t*** **value)**
	3 ×3 mm	7.07 ± 0.00	6.75 ± 1.05	7.04 ± 0.07	0.031^*^/2.221	0.007^**^/2.833	0.048^*^/−2.026
	6 ×6 mm	28.26 ± 0.06	27.31 ± 2.60	28.08 ± 0.55	0.009^**^/2.731	0.026^*^/2.297	0.035^*^/−2.155
	9 ×9 mm	63.12 ± 0.71	60.39 ± 5.36	62.69 ± 1.80	0.000^***^/3.738	0.118/1.517	0.004^**^/−2.982
	12 ×12 mm	109.76 ± 1.05	104.46 ± 7.97	108.97 ± 2.53	0.000^***^/4.520	0.063/2.006	0.001^***^/−3.674
	6–3 mm^2^	21.20 ± 0.06	20.55 ± 1.77	21.04 ± 0.53	0.009^**^/2.694	0.047^*^/2.039	0.056/−1.837
	9–6 mm^2^	34.85 ± 0.71	33.08 ± 3.30	31.99 ± 12.58	0.000^***^/3.880	0.122/1.621	0.537/0.620
**CVI**		***n*** **=** **51**	***n*** **=** **53**	***n*** **=** **71**	**(*****p/t*** **value)**	**(*****p/t*** **value)**	**(*****p/t*** **value)**
	3 ×3 mm	0.29 ± 0.09	0.32 ± 0.12	0.30 ± 0.10	0.160/−1.417	0.517/−0.649	0.373/0.894
	6 ×6 mm	0.26 ± 0.07	0.27 ± 0.09	0.26 ± 0.09	0.302/−1.038	0.941/−0.075	0.338/0.962
	9 ×9 mm	0.24 ± 0.06	0.23 ± 0.07	0.23 ± 0.08	0.802/0.251	0.358/0.922	0.525/0.638
	12 ×12 mm	0.24 ± 0.05	0.21 ± 0.06	0.22 ± 0.07	0.050^*^/ 1.983	0.072/1.732	0.817/−0.232
	6–3 mm^2^	0.03 ± 0.04	0.04 ± 0.05	0.04 ± 0.03	0.122/−1.560	0.037^*^/−2.397	0.954/0.057
	9–6 mm^2^	0.02 ± 0.02	0.04 ± 0.03	0.04 ± 0.06	0.000^***^/−3.80	0.025^*^/−2.275	0.029^*^/−2.207
**CVV**		***n*** **=** **51**	***n*** **=** **53**	***n*** **=** **71**	**(*****p/t*** **value)**	**(*****p/t*** **value)**	**(*****p/t*** **value)**
	3 ×3 mm	0.70 ± 0.32	0.85 ± 0.41	0.75 ± 0.39	0.033^*^/−2.167	0.377/−0.886	0.186/1.330
	6 ×6 mm	2.42 ± 1.01	2.79 ± 1.28	2.49 ± 1.30	0.103/−1.638	0.764/−0.289	0.192/1.312
	9 ×9 mm	4.87 ± 1.91	5.10 ± 2.27	4.66 ± 2.39	0.569/−0.571	0.598/0.512	0.301/1.038
	12 ×12 mm	8.18 ± 2.84	7.71 ± 3.47	7.49 ± 3.63	0.469/0.728	0.254/1.108	0.751/0.318
	6–3 mm^2^	1.73 ± 0.71	1.94 ± 0.89	1.73 ± 0.93	0.177/−1.360	0.979/−0.025	0.274/1.100
	9–6 mm^2^	2.45 ± 0.96	2.31 ± 1.07	2.04 ± 1.50	0.497/0.681	0.095/1.6801	0.629/0.484
**CT**		***n*** **=** **51**	***n*** **=** **53**	***n*** **=** **69**	**(*****p/t*** **value)**	**(*****p/t*** **value)**	**(*****p/t*** **value)**
	3 ×3 mm	339.28 ± 119.25	362.12 ± 91.49	337.55 ± 101.67	0.275/−1.098	0.932/0.086	0.170/1.382
	6 ×6 mm	325.08 ± 113	338.03 ± 85.85	316 ± 93.09	0.511/−0.66	0.631/0.482	0.183/1.34
	9 ×9 mm	302.97 ± 98.63	308.23 ± 78.9	291.35 ± 82.16	0.764/−0.301	0.487/0.697	0.258/1.137
	12 ×12 mm	288.76 ± 84.44	283.52 ± 74.39	274.88 ± 72.16	0.751/0.318	0.351/0.937	0.546/0.606
	6–3 mm^2^	14.21 ± 16.08	24.1 ± 16.09	21.55 ± 18.6	0.002^**^/−3.135	0.026^*^/−2.312	0.428/0.795
	9–6 mm^2^	22.11 ± 19.26	29.8 ± 12.98	33.09 ± 60.42	0.018^*^/−2.378	0.213/−1.251	0.661/−0.39

* *p ≤ 0.05, there is a statistical difference*;

** *p ≤ 0.01*,

*** *p ≤ 0.001, there is a significant difference*.

Additionally, the mean CP values were significantly decreased in both NPDR and PDR groups, compared to that of control subjects. Importantly, the rate of CP was lower in the PDR group than that in the NPDR group in terms of captures in 3 mm (3 ×3 mm), 6 mm (6 ×6 mm), 9 mm (9 × 9 mm). Thus, the rate of CP was significantly decreased in the diabetic eyes of PDR and NPDR patients, and the intensity of reduction in the rate of CP was directly linked to the progression of DR pathology from NPDR to PDR stage.

Subsequently, the PDR group exhibited a significantly different mean value of CVI at 9–6 mm^2^ compared to NPDR group. However, there was only difference in the 3 mm (3 × 3 mm) captures of CVV between the control subjects and PDR patients (0.70 ± 0.32 vs. 0.85 ± 0.41; *p* = 0.033).

## Discussion

Previous study has shown that patients with T2DM are more likely to have enlarged FAZ compared to their age-matched non-diabetic control subjects (20). In diabetic patients eyes, DR can lead to progressive macular ischemic changes. Under this circumstance, the advent of SS-OCTA technology provides a unique opportunity to non-invasively study macular perfusion and several other pathological parameters in the diabetic eye (20–22). Our patho-clinical observations confirmed gradually increasing FAZ and RT, while the significant overall decrease in VD in all areas of central fovea and PD as the DR pathology moved forward from NPDR to PDR stage (6, 23, 24). The rapid development of SS-OCTA technology provided an accurate, fast, and reproducible method for detecting fundus VD modulations in DR patients.

Compared with OCTA, the SS-OCTA provides superior angiogram montages covering larger areas with distinct retinal capillaries to the mid-periphery (25, 26). It is beyond the scope of the traditional seven-field Early Treatment Diabetic Retinopathy Study (ETDRS) standard. Therefore, SS-OCTA analysis yields high-content information for disease diagnosis. By employing SS-OCTA technique, we found significant differences annular area of choroidal blood perfusion, large and medium vessel indices, and choroidal thickness between the healthy control group and PDR or NPDR group. It suggested that the retinal imaging of SS-OCTA may reveal crucial findings facilitating DR diagnosis and understand the patho-mechanisms of NPDR to PDR progression.

Furthermore, we observed that both NPDR and PDR patients exhibited considerable ischemic alterations in the macular regions, in addition to the decreased retinal VD and PD around the central fovea. This observation indicated that the change of retinal ischemic area in DR patients might not be limited on the macular area. This is similar to previous studies (27). Additionally, CVI and CVV are novel quantitative parameters for choroidal choroidal vascular health markers, suggesting severe choroidal injury in PDR. Similar to previous reports (28, 29), there were no significant differences in CVI and CVV of fovea between the control subjects and NPDR group. However, this were contradictory about the correlation between CVI or CVV in the control subjects and PDR group, possibly because we did not distinguish whether the patients also had DME (30). Although the algorithm of SS-OCTA for detecting choroidal vascular correlation index still needs to be refined, SS-OCTA makes it possible for us to evaluate choroidal quantification, which will provide advantages for the diagnosis and treatment of choroidal vascular diseases in different DR patients.

Notably, the difference in the ischemic damage of macular area between the DR patients and control subjects was found to be directly associated with the area measured in the annular region with the fovea at the center. While, based on these findings, it was obvious that there would be a significant difference in macular lesion area between NPDR and PDR patients. On the other hand, a comparatively larger lesion area might be helpful in providing a more accurate diagnosis in the case of PDR than in NPDR. Subsequently, we identified differential damage intensities in peripheral retinal perfusion rates between the PDR and NPDR patients, as well as with the healthy control group, which might provide a new paradigm shift in our understanding of the mechanism of the progression of DR. Together, these findings demonstrated the potential benefits of SS-OCTA evaluation for diagnostic stratification between different stages of retinal injury.

Although SS-OCTA devices provide the possibility of wide-field imaging, there are still some limitations. For example the artifacts caused by the eye movement or blinking, the segmentation errors, and so on. And the leakage could not be detected by SS-OCTA. Additionally, a controlled prospective study with larger sample size was needed to further study.

In summary, identification of the differences in retinal VD and RT between NPDR and PDR patients could indicate possible mechanistic of DR progression. SS-OCTA examination has promising benefits in the early diagnosis of DR pathology as well as in the differentiation between various stages of disease progression.

## Data Availability Statement

The original contributionspresented in the study are included in the article/Supplementary Material, further inquiries can be directed to the corresponding author/s.

## Ethics Statement

The Human Research was approved by the Institutional Ethics Committee of Shandongeye hospital (Approval No. 2019S001) on November 11, 2019. The patients/participants provided their written informed consent to participate in this study. Written informed consent was obtained from the individual(s) for the publication of any potentially identifiable images or data included in this article.

## Author Contributions

TiL, QZ, and WLdesigned and conducted clinical examinations, wrote, and revised the manuscript. GS, WW, MF, XX, and ToL prepared the figures and tables and interpreted the results. All authors contributed to the revision steps and approved the final version of the manuscript for submission.

## Funding

This studywas supported by a grant from the Natural Key Research and Development Project (2016YFC1305500), Bethune Langmu Young Scholars Research Fund (BJ-LM2021007J).

## Conflict of Interest

The authors declare that the research was conducted in the absence of any commercial or financial relationships that could be construed as a potential conflict of interest.

## Publisher's Note

All claims expressed in this article are solely those of the authors and do not necessarily represent those of their affiliated organizations, or those of the publisher, the editors and the reviewers. Any product that may be evaluated in this article, or claim that may be made by its manufacturer, is not guaranteed or endorsed by the publisher.
