# Supplementary material for: Retinal and Choroidal Vascular Perfusion and Thickness Measurement in Diabetic Retinopathy Patients by the Swept-Source Optical Coherence Tomography Angiography
Source: Front Med (Lausanne). 2022 Mar 18;9:786708. doi: 10.3389/fmed.2022.786708 (PMC8971655; doi:10.3389/fmed.2022.786708)
Supplement: Supplementary file 1 [file Presentation_1.pdf]

Supplementary materials

Retinal and choroidal vascular perfusion and thickness measurement in diabetic retinopathy patients by the swept-source optical coherence tomography angiography

Tingting Liu [1,2,3,4*†], Wei Lin[5†], Genggeng Shi [1,2,3], Wenqi Wang [6], Meng Feng[5], Xiao Xie [6], Tong Liu [7] and Qingjun Zhou [2,3,4,8*]

[1] Eye Hospital of Shandong First Medical University (Shandong Eye Hospital), Jinan, China.

[2] State Key Laboratory Cultivation Base, Shandong Provincial Key Laboratory of Ophthalmology, Shandong Eye Institute, Qingdao, China.

[3] Shandong First Medical University & Shandong Academy of Medical Sciences, Qingdao, China.

[4] School of Ophthalmology, Shandong First Medical University, Jinan, China.

[5] School of Basic Medicine, Shandong First Medical University and Shandong Academy of Medical Science, Jinan, China.

[6] The first clinical medical college of Shandong University of Traditional Chinese Medicine, Jinan, China.

[7] Departments of Medicine, Xizang Minzu University, Xianyang, China.

[8] Qingdao Eye Hospital of Shandong First Medical University, Qingdao, China

[*]corresponding author:

Tingting Liu, PhD

Eye Hospital of Shandong First Medical University (Shandong Eye Hospital), Jinan 250002, China.

Email: tingtingliu@vip.sina.com.

Qingjun Zhou, PhD

Qingdao Eye Hospital of Shandong First Medical University, Qingdao 266071, China.

Email: qjzhou2000@126.com.

[†] co-first author.

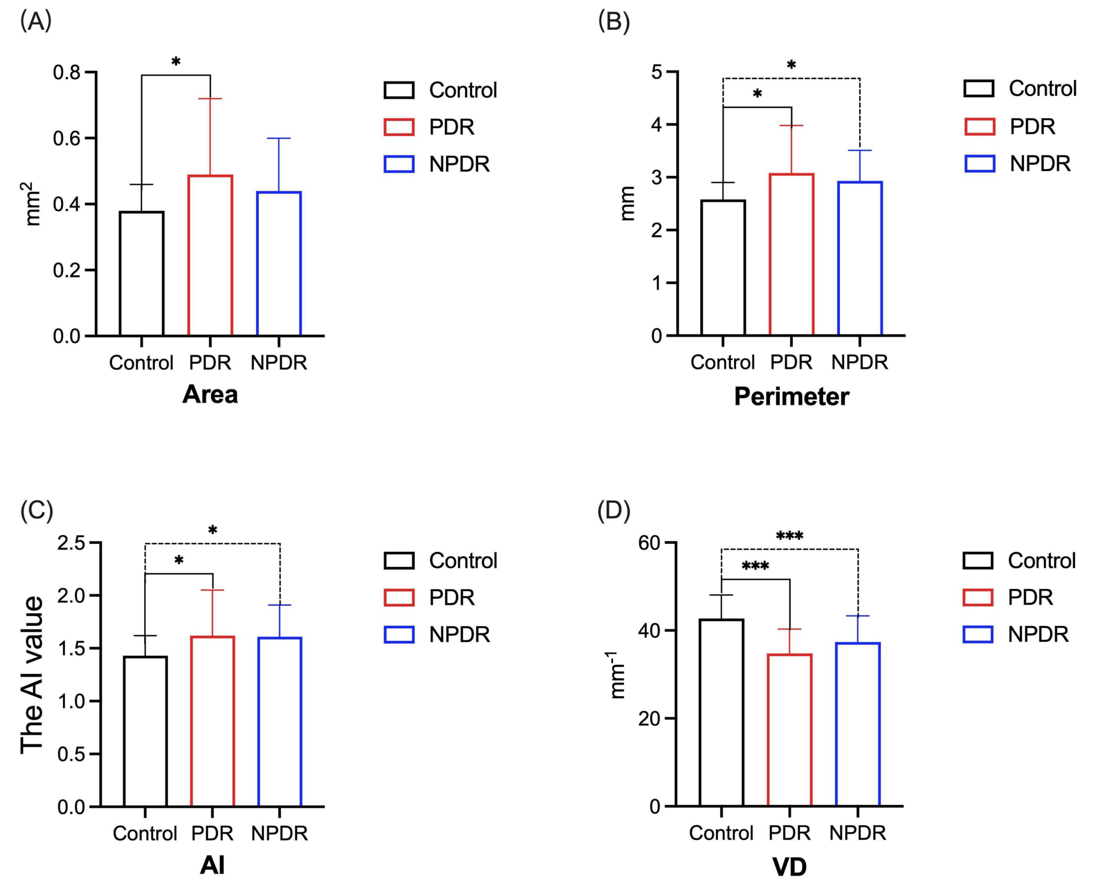

Figure S1. The changes of fovea FAZ in healthy control group, PDR group and NPDR group by SS-OCTA(*$p \leq 0.05$, there is a statistical difference.**$p \leq 0.01$, ***$p \leq 0.001$, there is a significant difference).

(A) Comparison of the FAZ area in the healthy controls, PDR and NPDR groups.

(B) Comparison of the FAZ perimeter in the healthy controls, PDR and NPDR groups.

(C) Comparison of AI in the healthy controls, PDR and NPDR groups. The AI value is calculated as $4\pi \times$FAZ area/perimeter^2.

(D) Comparison of VD in the healthy controls, PDR and NPDR groups.

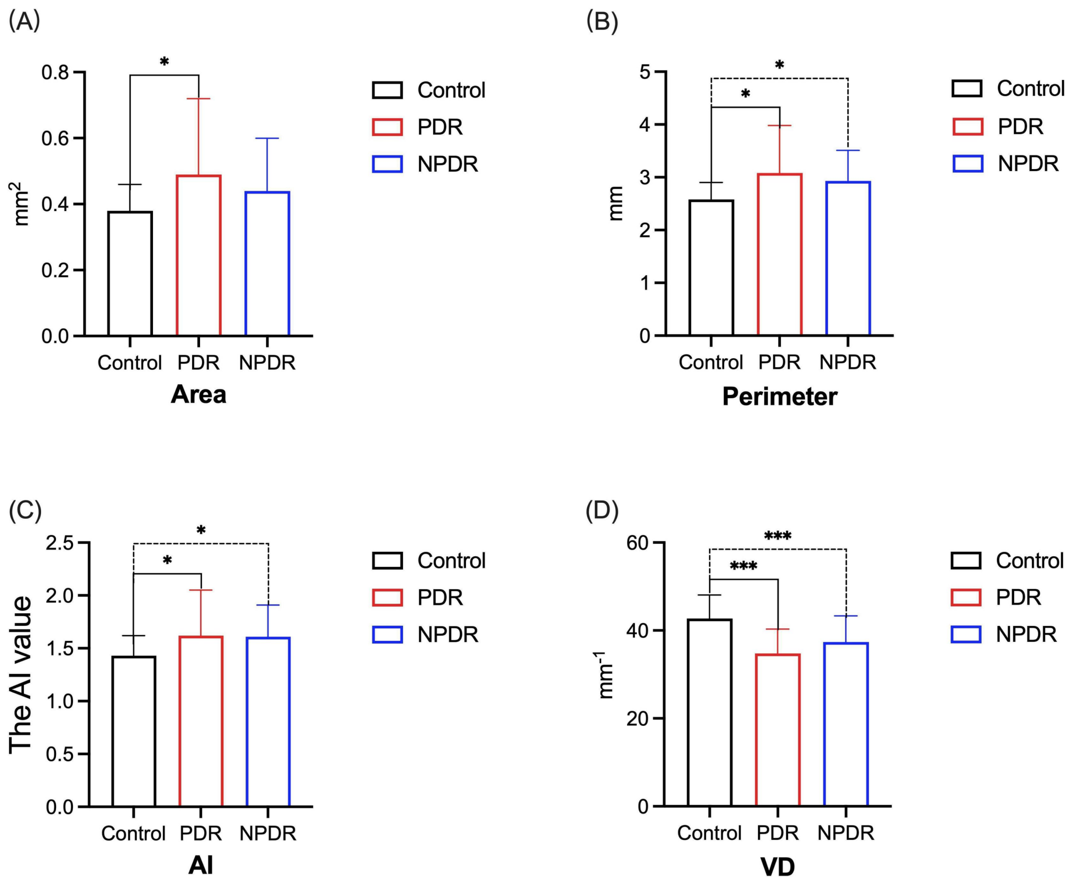

Figure S2. The changes of the choroidal vascular complexes in healthy control group, PDR group and NPDR group by SS-OCTA(*$p \le 0.05$, there is a statistical difference.**$p \le 0.01$, ***$p \le 0.001$, there is a significant difference).

(A) Comparison of CP in 3×3(mm), 6×6(mm), 9×9(mm), 12×12(mm), 3-6(mm^2) and 6-9mm (mm^2) in the healthy controls, PDR and NPDR groups.

(B) Comparison of CVI index in 3×3(mm), 6×6(mm), 9×9(mm), 12×12(mm), 3-6(mm^2) and 6-9mm (mm^2) in the healthy controls, PDR and NPDR groups. The ratio is the ratio of choroidal vascular luminal volume to the total choroidal volume.

(C) Comparison of CVV in 3×3(mm), 6×6(mm), 9×9(mm), 12×12(mm), 3-6(mm^2) and 6-9mm (mm^2) in the healthy controls, PDR and NPDR groups.

(D) Comparison of CT in 3×3(mm), 6×6(mm), 9×9(mm), 12×12(mm), 3-6(mm^2) and 6-9mm (mm^2) in the healthy controls, PDR and NPDR groups.
